# Barcoding Melting Curve Analysis for Rapid, Sensitive, and Discriminating Authentication of Saffron (*Crocus sativus* L.) from Its Adulterants

**DOI:** 10.1155/2014/809037

**Published:** 2014-10-30

**Authors:** Chao Jiang, Liang Cao, Yuan Yuan, Min Chen, Yan Jin, Luqi Huang

**Affiliations:** ^1^Beijing Key Laboratory of Protection and Application of Chinese Medicinal Resources, Beijing Normal University, Beijing 100875, China; ^2^State Key Laboratory Breeding Base of Dao-di Herbs, National Resource Center for Chinese Materia Medica, China Academy of Chinese Medical Sciences, Beijing 100700, China; ^3^Institute of Agricultural and Biological Resources Utilization, Hunan Academy of Agricultural Sciences, Changsha 410125, China

## Abstract

Saffron (*Crocus sativus* L.) is one of the most important and expensive medicinal spice products in the world. Because of its high market value and premium price, saffron is often adulterated through the incorporation of other materials, such as *Carthamus tinctorius* L. and *Calendula officinalis* L. flowers, *Hemerocallis* L. petals, *Daucus carota* L. fleshy root, *Curcuma longa* L. rhizomes, *Zea may* L., and *Nelumbo nucifera* Gaertn. stigmas. To develop a straightforward, nonsequencing method for rapid, sensitive, and discriminating detection of these adulterants in traded saffron, we report here the application of a barcoding melting curve analysis method (Bar-MCA) that uses the universal chloroplast plant DNA barcoding region *trnH-psbA* to identify adulterants. When amplified at DNA concentrations and annealing temperatures optimized for the curve analysis, peaks were formed at specific locations for saffron (81.92°C) and the adulterants: *D. carota* (81.60°C), *C. tinctorius* (80.10°C), *C. officinalis* (79.92°C), *Dendranthema morifolium* (Ramat.) Tzvel. (79.62°C), *N. nucifera* (80.58°C), *Hemerocallis fulva* (L.) L. (84.78°C), and *Z. mays* (84.33°C). The constructed melting curves for saffron and its adulterants have significantly different peak locations or shapes. In conclusion, Bar-MCA could be a faster and more cost-effective method to authenticate saffron and detect its adulterants.

## 1. Introduction

*Crocus sativus* L. is a perennial triploid sterile herb of the family Iridaceae that has purple flowers and 3-branched style [1]. Red-dried stigmas of this herb (saffron) are extensively employed in cooking, in the preparation of saffron dye, and in Oriental medicine. Saffron has been used in the Mediterranean region for thousands of years. Recent pharmaceutical research has indicated that saffron may have beneficial anticancer, antioxidant, analgesic, anti-inflammatory, and antidepressant properties [2–6]. Saffron is commonly considered the most expensive medicinal spice due to its high demand and low supply. Approximately 75,000 crocus blossoms or 225,000 handpicked stigmas are required to produce one pound of this unique spice [7]. In 2009, the market price for 1 kg of quality saffron spice exceeded $5,000 [8]. The worldwide market for saffron is worth nearly $1 billion. Although the demand for saffron in the Middle East and West is increasing, the amount of globally produced dried saffron has remained constant at approximately 300 ton per year, and production has even decreased rapidly in many countries that have traditionally produced saffron [9].

Due to its high market value, perceived value, demanding production, and premium price, attempts have been made to adulterate saffron with various substances with similar color and morphology to increase the volume and weight of commercial lots. The most frequently incorporated materials are* Carthamus tinctorius* L. and* Calendula officinalis* L. flowers,* Hemerocallis* L. petals,* Daucus carota* L. fleshy root,* Curcuma longa* rhizomes,* Zea may* L., and* Nelumbo nucifera* Gaertn. stigmas [10–12]. The methods for determining adulteration in saffron include traditional pharmacognostic analysis, chromatographic and spectroscopic approaches, physical techniques, and molecular markers. Pharmacognostic analysis of morphological traits, such as starch granules, epidermal stigma residues, pollen granules, and upper-end stigma residues with epitheliums, is often experience-dependent and extremely time consuming when screening a large number of samples [13]. Chemical analytical methods include UV-Vis spectrophotometry [14, 15], high-performance liquid chromatography [16, 17], near- or middle-infrared spectroscopy [18, 19], and nonaqueous capillary electrophoresis [20]. These methods are based on measuring the amount of picrocrocin, safranal, and crocin in saffron. These approaches have been viewed as viable candidates for a more reliable evaluation of saffron purity. The quantity of bioactives, such as safranal and crocin, in saffron is strongly affected by the geographic origin, drying methods, and storage [17, 18, 21–23]. The sensitivity of many analytical methods is commonly low, and these methods cannot sufficiently distinguish natural saffron from its misused substitutes [24, 25]. For example, the UV-Vis spectrometric method (ISO/TS 3632-2) as an ISO standard for saffron authentication was unable to detect the addition of up to 10–20% (w/w) of the saffron adulterants* Calendula*,* Carthamus* flowers, or ground turmeric rhizome. The addition of calibrated amounts of other plant materials rich in carotenoids may easily go undetected [13, 26]. Therefore, chemical methods to detect adulterants, particularly low levels, in commercial saffron are not sufficient.

Molecular markers can detect differences at the DNA level and offer numerous advantages over conventional phenotype-based alternatives because they are stable and detectable in all tissue types, regardless of the growth environment, development state, or differentiation status [27]. The advantages of this approach include high sample throughput, low detection limit, and good interlaboratory reproducibility. As techniques develop, DNA-based methods could be used in the analysis of processed foodstuff, patent drugs, fixed paraffin, adequate extraction, or vegetable oil [28–30]. Thus, molecular markers offer several advantages for the determination of food authenticity in routine quality control [31, 32]. Several PCR-based methods have been used to detect saffron adulteration, including allele-specific PCR (AS-PCR) [33], DNA sequence analysis [10, 33, 34], and RAPD-derived SCAR markers [12, 13, 23, 25]. AS-PCR and SCAR markers were derived from specific fragments. These markers require screening new fragments or alleles to design specific primers for any new or unfamiliar adulterants, which limits its application in the detection of new adulterants. Thus, new molecular markers, particularly DNA barcode-based universal primers methods, have been proposed and rapidly developed.

DNA barcodes are short orthologous DNA sequences that are used to identify species. By using a standardized DNA region as a tag, DNA barcodes provide a rapid, accurate, and automatable identification method. It has been widely used in plant identification, biodiversity assessment and conservation, adulteration detection, and traditional medicine authentication [35–37]. Different plastid DNA regions, including the* rbc*L,* mat*K,* rpo*B, and* rpo*C1 genes, the noncoding* atp*F-*atp*H,* psb*K-*psb*I,* trnH-psbA,* and* trnL-F* spacers, and the nuclear internal transcribed spacer (ITS2), have been proposed to serve as DNA barcodes for identifying flowering plants [38, 39]. The barcodes* rbc*L,* mat*K, and* trnH-psbA* and ITS2 fragments have been recommended by CBOL (Consortium for the Barcode of Life) for the barcoding of seed plants and were successful at discriminating more than 79 percent of plant groups [39]. Despite these advantages, this technique requires post-PCR processing, DNA sequencing, sequence alignment, and sequence analysis with the program BlastN or the construction of phylogenetic trees. These steps make DNA barcoding relatively complicated, time consuming, and unintuitive.

Melting curve analysis is a fast and sensitive method for differentiating PCR production by fluorescence monitoring of the melting curve of the double-stranded DNA that is intercalated by the dye SYBR Green I in a real-time PCR system [40]. Melting curve analysis has many advantages (e.g., gel-free, quick, and inexpensive). Results can be obtained without additional post-PCR processing in less than 2 h. These characteristics make it widely used in pathogenic identification [41], food authenticity [42], and biological diagnostics [43].

Herein, we describe a new application of SYBR Green melting curve analysis that is coupled with DNA barcoding and uses universal regions for the rapid detection and adulteration measurement of saffron and their commercial food products. Bar-MCA distinguished saffron and its different adulterants species and detected trace adulterants in commercial products.

## 2. Materials and Methods

### 2.1. Plant Material and DNA Isolation

Eight dried commercial* C. sativus* samples were purchased from herb markets in China, including two samples from the Anguo herb market, one sample from the Bozhou herb market, and five batches from the TongRenTang pharmacy. Three* C. sativus* samples marketed as whole stigmas were purchased from Iran. Another six unidentified commercial samples were collected from the Shanghai Traditional Chinese Medicine Co. Ltd. (Shanghai, China) and the Anguo herb market (Anguo, China). Twenty-two adulteration samples, such as* D. morifolium*,* C. officinalis*,* C. tinctorius*,* N. nucifera*,* H. fulva*, and* Z. mays*, were purchased from herb markets, as described in Table 1. Fresh* D. carota* and* Z. may* were obtained from a supermarket and frozen at −80°C for DNA extraction. All of the collected samples were identified by a taxonomist, except for 6 unidentified* C. sativus* commercial samples (Table 1).

Plant materials were frozen in liquid nitrogen and ground to a fine powder by a Retsch MM 400 Mixer Mill (Retsch Technology GmbH, Haan, Germany). The total genomic DNA was isolated from fresh and dried material as previously described using the modified CTAB method [34]. The extracted DNA was quantified by optical absorbance at 260 nm. For the adulteration detection, each sample consisted of at least 0.5 g of powdered material, and 50 mg of powder was randomly selected for DNA extraction.

### 2.2. Real-Time PCR Amplification and Melting Curve Analysis

PCR amplification, DNA melting, and fluorescence signal collection were performed in a total volume of 20 *μ*L on an ABI 7500 real-time PCR system (Applied Biosystems, California, USA). The reaction mixture contained 10 ng of genomic DNA, 10 *μ*L of 2x SYBR Green Premix Ex Taq (Takara Bio Group, Kyoto, Japan), 0.2 *μ*L of 10 mM forward and reverse primers (synthesized by Beijing Genomics Institute, Beijing, China), and 0.4 *μ*L of 50x ROX reference Dye II (Takara Bio Group, Kyoto, Japan). The five pairs of candidate barcoding primers and their real-time PCR reaction conditions are shown in Table 2. Melting curves were generated after the last extension step. The temperature was increased from 60 to 95°C at 0.1°C/s. The melting curves were analyzed with the ABI 7500 version 2.0 software (Applied Biosystems, California, USA).

### 2.3. Primer Screening

Four CBOL-recommend DNA barcoding fragments (*trnH-psbA*,* rbc*L,* mat*K, and ITS2) and a* trnL-F* spacer fragment were selected to authenticate saffron and its adulterants by analysis of the melting curve shapes and the melting temperature (*T*_*m*_) of the amplicons. The barcoding fragments were amplified with the primer pairs psbAF/trnHR, 1F/724R, 3F/1R, ITS2/ITS3, and trnL/trnF, respectively. The fragments that could discriminate between saffron and all of its adulterants were selected for further analysis. The primer sequences and PCR conditions are shown in Table 2.

### 2.4. Optimizing the Bar-MCA Conditions

The influence of the annealing temperature, PCR cycle number, and DNA template concentration on melting curve shape and *T*_*m*_ was investigated. Every experiment was repeated 3 times. The optimized Bar-MCA conditions were selected to construct the melting curve models.

### 2.5. Construction of the Melting Curve Model for Saffron

Eleven samples of saffron were selected to construct the melting curve models of saffron. The fluorescence melting curve was monitored using the appropriate primers and optimized conditions. The ABI 7500 version 2.0.1 software was employed for the analysis and construction of the melting curve models of saffron. The precision and reproducibility of melting curve models were also investigated to study the method's accuracy and stability.

### 2.6. PCR and Sequence Analysis

The botanical origins of all authentication samples were confirmed by amplification and sequencing of the* trnH-psbA* fragment. PCR was performed in a 25-*μ*L reaction mixture containing 10 ng of genomic DNA, 2.5 *μ*L of 10x Ex Taq (Takara Bio Group, Kyoto, Japan), 0.2 *μ*L of 10 *μ*M forward and reverse primers, 1 *μ*L of 10 mM dNTP stock, and 1 unit of Ex Taq polymerase (Takara Bio Group, Kyoto, Japan). PCR amplification was performed in a GeneAmp PCR 9700 system (Applied Biosystems, California, USA) programmed for 5 min at 95°C, 35 cycles of 30 s at 95°C, 30 s at 58°C, and 40 s at 72°C and a final extension for 5 min at 72°C. The PCR products were confirmed by electrophoresis in a 1.5% agarose gel and directly visualized with ethidium bromide under UV light. The PCR products were directly sequenced in an automated ABI 3730 sequencer (Applied Biosystems, California, USA) by Beijing Genomics Institute. The sequences were aligned with the ClustalW program. The GC content and predicted melting temperatures were calculated by OligoCalc, an online program [44].

Amplified* trnH-psbA* fragments of the unidentified sample CsA1 were subcloned into the pMD19-T vector (Takara Bio Group, Kyoto, Japan) for sequencing, and 3 positive clones were screened and sequencing using an ABI 3730 sequencer. Sequences were identified by the web-based MegaBLAST algorithm in the NCBI nucleotide nr/nt database by the default settings.

## 3. Results and Analysis

### 3.1. Primers Screening

**Figure 1:**
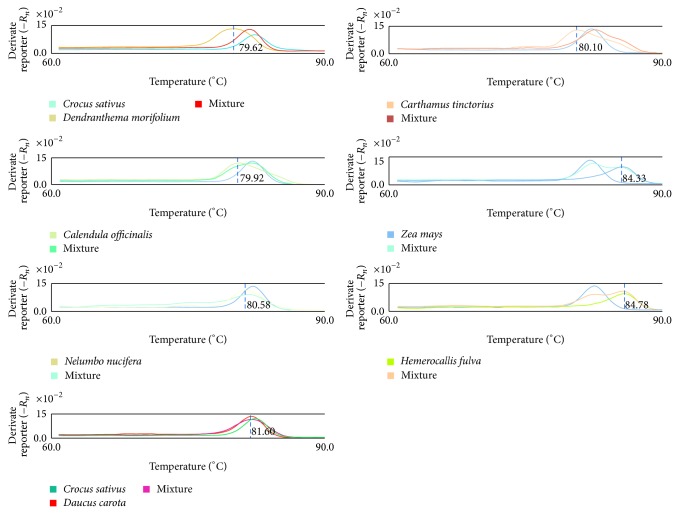
Melting curves of saffron and its adulterants. Saffron, 7 adulterants, and 1 : 1 mix of authentic saffron with adulterants were amplified in triplicate by the primer pair psbAF/trnHR and performed melting curve analysis by a real-time PCR system. Mixture means mix of saffron with the corresponding adulterant in one PCR reaction.

The melting curve is influenced by the DNA GC content, length, and sequence arrangement. Polymorphisms of barcode regions among saffron and its adulterants could affect the melting curve and melting temperature. To obtain suitable primers for authentication, 5 barcoding primers for the amplification plastid DNA regions* rbc*L,* mat*K genes, the noncoding* trnH-psbA*, the* trnL-F* spacer, and the nuclear internal transcribed spacer (ITS2) were used for the amplification of saffron and its adulterants by a rapid real-time PCR procedure. The melting curves were analyzed by the ABI 7500 version 1.4 software. The results show that the melting curves of saffron and its adulterants generated by psbAF/trnHR, 1F/724R, ITS2/ITS3, 3F/1R, and trnL/trnF were all single peaks. The difference between the *T*_*m*_ value of saffron and that of its adulterants ranged from 0.6 for the primer pair ITS2/ITS3 to 1.2 for psbAF/trnHR. When amplified by the primer pair psbAF/trnHR, distinct melting curves and *T*_*m*_ values were found for* D. morifolium*,* C. officinalis*,* C. tinctorius*,* N. nucifera*,* H. fulva*,* Z. mays*,* D. carota*, and saffron (Figure 1). Thus, this primer was selected as the optimal primer for the authentication tests.

### 3.2. Influence of Annealing Temperature and DNA Concentration

**Figure 2:**
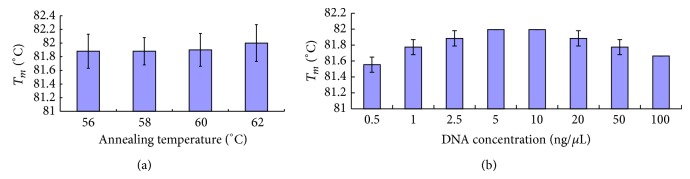
Influence of annealing temperature and DNA concentration on saffron melting temperature. (a) Annealing temperature. PCR was performed on different annealing temperature (56, 58, 60, and 62°C) in triplicate. (b) DNA concentration. Saffron DNA was serially diluted, and each concentration of saffron DNA was amplified in triplicate by the primer pair psbAF/trnHR.

To perform a melting curve analysis, the target product must be amplified by real-time PCR to acquire a sufficient amount of PCR product to combine with the fluorescent dye SYBR Green I. Factors that affect the PCR process, such as annealing temperature and DNA concentration, could influence the melting curve results. To study the influence of annealing temperature on the saffron melting curve, 3 distinct saffron samples were amplified by the primer pair psbAF/trnHR in triplicate. Table 2 shows the PCR conditions. The annealing temperature was set to 56, 58, 60, and 62°C. The melting curves were collected. The curve shapes and *T*_*m*_ results were compared for different annealing temperatures. The results indicate that when annealing at 56, 58, 60, and 62°C, no differences were found in the melting curves, and the *T*_*m*_ ranged from 81.88 ± 0.25 to 82.00 ± 0.27 (Figure 2(a)). The ANOVA analysis showed no significant differences between the different annealing temperatures. Because the smallest standard deviation (SD = ±0.20) was at 58°C, this temperature was selected as the optimal annealing temperature (Figure 2(a)).

The effect of DNA concentration on the melting curve was also considered. Saffron DNA was randomly selected. The concentration was adjusted to 100 ng/*μ*L and serially diluted to 100, 50, 20, 10, 5, 2.5, 1, and 0.5 ng/*μ*L. Each concentration of saffron DNA was amplified in triplicate by the primer pair psbAF/trnHR. The annealing temperature was 58°C, and the cycle number was 40. The curve shape and *T*_*m*_ of saffron were analyzed by ABI 7500 version 1.4 software. The results indicated that the DNA concentration affects the saffron melting curve. DNA concentrations that are too high or too low cause the *T*_*m*_ value to decrease and the standard deviation to increase (Figure 2(b)). No significant differences (*P* > 0.05) were found for the saffron melting curve when the DNA concentration ranged from 2.5 to 20 ng/*μ*L. The melting curve was not different when the DNA concentration was 5–10 ng/*μ*L. This concentration was selected as the optimal DNA concentration.

### 3.3. Constructing the Melting Curve Model of Saffron

**Figure 3:**
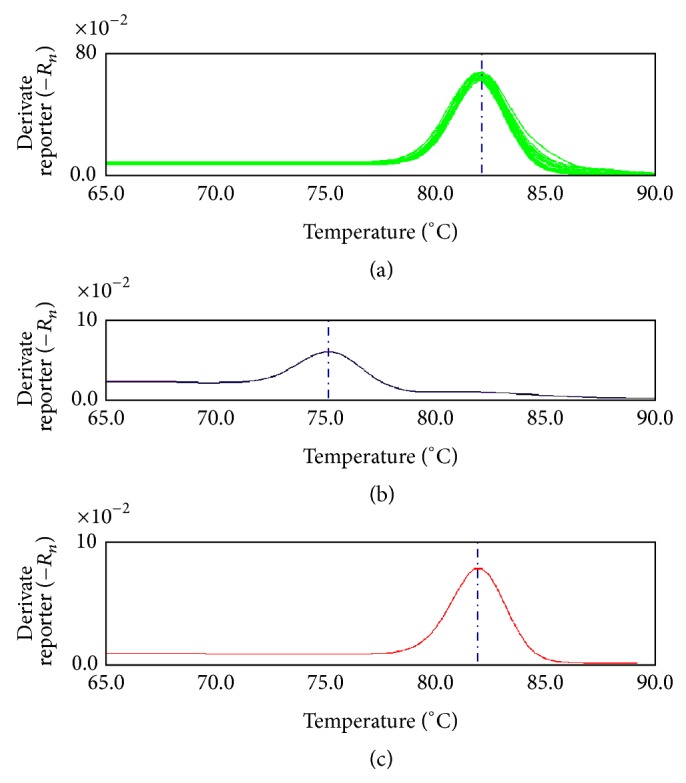
Melt curve model of* Crocus sativus* by* trnH-psbA* barcode fragment. (a) Melt curve 11* Crocus sativus* samples; (b) negative control result of Bar-MCA by replacing DNA temple by ddH_2_O. (c) Melt curve model of* Crocus sativus* based on 11* Crocus sativus* samples.

Eleven batches of saffron were randomly selected. The DNA from these samples was amplified in triplicate by the primer pair psbAF/trnHR. The melting curves of the PCR products were analyzed and used to construct the melting curve models. The 33 saffron* trnH-psbA* PCR products had the same sharp melting curve, and the average *T*_*m*_ of the saffron* trnH-psbA* fragment was 81.92°C, with a standard deviation of 0.20 and relative standard deviation (RSD) of 0.24% (Figure 3). To test the precision of the saffron melting curve analysis, saffron DNA was randomly selected and amplified by the primer pair psbAF/trnHR for melting curve analysis and repeated 6 times. The results show that the 6 melting curves had the same shape and sharpness. The *T*_*m*_ of the saffron melting curve was 81.90 ± 0.063°C, and the RSD was 0.077%. To verify the reproducibility, a saffron sample was divided into 6 aliquots, and the melting curve analysis was performed. The saffron melting curves were similar, with an averaged *T*_*m*_ of 81.90 ± 0.090°C and an RSD of 0.11%. A maximum difference of 0.17°C was observed in the melting peak temperatures between different runs and a maximum difference of 0.12°C was observed within the same run.

### 3.4. Bar-MCA to Authenticate Saffron

The PCR products of the* trnH-psbA* fragment of* D. morifolium*,* C. officinalis*,* C. tinctorius*,* N. nucifera*,* H. fulva*,* Z. mays*, and* D. carota* were also amplified by the primers psbAF/trnHR. The PCR conditions are described in Table 2. The melting curves were acquired with ABI 7500 real-time PCR systems, and the melting curve models were constructed for each adulterant. To test the capability of the curve melting to distinguish samples, the DNA of saffron was admixed with the same concentration of the DNA of each of its adulterants, and the melting curve of the* trnH-psbA* fragment of each mixture was also acquired and analyzed. The resulting melting curve shapes and *T*_*m*_ values of saffron, its adulterants, and the mixtures were significantly different. These peaks formed at 81.92°C for saffron, 81.60°C for* D. carota*, 80.10°C for* C. tinctorius*, 79.92°C for* C. officinalis*, 79.62°C for* D. morifolium*, 80.58°C for* N. nucifera*, 84.78°C for* H. fulva*, and 84.33°C for* Z. mays* (Figure 1). The melting curve analysis of the amplified DNA had sufficient sensitivity and specificity to differentiate saffron and its adulterants or its mixtures based on the *T*_*m*_ differences coupled with the sharpness of the melting curves (Figure 1). The adulterants could be distinguished on a species level. The differences in *T*_*m*_ value could be used as a simple biomarker to authenticate saffron and identify its adulterants. The results also show that psbAF/trnHR is a desired primer pair and that the polymorphisms within the* trnH-psbA* region among the eight species are sufficient to distinguish the adulterants.

### 3.5. Application of Bar-MCA for the Authentication of Unknown Commercial Saffron

**Figure 4:**
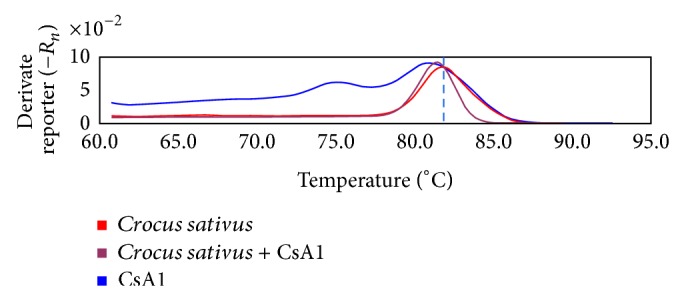
Bar-MCA for authenticate of unknown commercial saffron. Melting curve of authentic saffron (red line), unknown commercial sample (blue line), and its mixture (red-violet line) were significant different; this means the sample may be fraudulent.

To test the efficiency of the method for authenticating unknown commercial samples of saffron, 6 commercial saffron samples were tested using barcoding melting analysis with the psbAF/trnHR primers. The results show that 5 saffron samples from Beijing, Shanghai, and Anguo were authentic saffron and that 1 sample from the Anguo herb market (CsA1) was fraudulent (Figure 4). To confirm the results, CsA1 was mixed with the identified saffron, and the melting curve analysis was repeated. The melting curve of CsA1 (Figure 3 blue line) was different from the identified saffron (Figure 3, red line) and the mixture (Figure 3, red-violet line). Because the major peak of the CsA1 sample melting curve was located within the range of 80.5 to 81.6°C, only the *T*_*m*_ of the* D. carota* (81.60°C) and* N. nucifera* (80.58°C) melting curves fulfilled this characteristic. From this result, we can infer that CsA1 may be adulterated with* D. carota* or* N. nucifera*. However, because the melting curve depends on the GC content and length and sequence of the PCR product [40], the melting curve *T*_*m*_ will shift in a mixture sample (Figure 4, red-violet line). For a *T*_*m*_ lower than 80.5°C, incorporation with* C. tinctorius* (79.92°C) or* C. officinalis* (79.62°C) is probable. Under these circumstances, the sharpness of the melting curve can be used as supplementary means to authenticate saffron. The melting curve of the* N. nucifera trnH-psbA* fragment had a secondary peak at approximately 75°C. The unidentified sample CsA1 had the same secondary peak. The results imply that CsA1 may be adulterated with* N. nucifera*. To further confirm the results,* trnH-psbA* sequences retrieved from the unknown sample CsA1 were cloned into a PMD19-T vector for sequencing. Three positive clones were randomly selected and sequenced. MegaBLAST results indicate that the 2 clones were 100% identical with* C. sativus psb*A gene (GenBank: EU110147.1) and the 1 clone was similar to the* N. nucifera* complete chloroplast genome (GenBank: FJ754270.1) at 99% nucleotide similarity level. The agreement between the sequencing results and melting curve analysis confirms that the sample CsA1 was adulterated with* N. nucifera*.

## 4. Discussion

The precise identification of plant species is crucial for agriculture, industry, and consumers. Saffron is an important commercial medicinal plant that must be authenticated. However, the detection of fraudulent saffron is a challenging task because the physical, chemical, and classical morphological properties are not always easily identifiable, particularly when the sample is finely ground or when the spice is added to a seasoning mixture [13]. The present molecular authentication methods, such as AS-PCR, DNA sequence analysis, and SCAR markers, often require screening new fragments or alleles to design specific primers when new adulterants are encountered; thus, these techniques require time-consuming sequencing or have limited application. Our goal was to develop a reliable nonsequencing method based on melting curve analysis. We analyzed the melting curves and determined the *T*_*m*_ values for 5 barcode fragments of saffron and its adulterants. A primer that can discriminate between saffron and adulterants based on *T*_*m*_ was selected and evaluated using authentic saffron material at an optimized concentration and annealing temperature to determine the precision and reproducibility of the method.

Compared with current sequence-based species discrimination methods, melting curve analysis is a very promising technique, particularly in terms of costs and time. DNA melting was observed as a sudden decrease in the fluorescence of the dsDNA dye SYBR Green I as a sample was heated through the *T*_*m*_ of the product [40]. When the PCR products were denatured and annealed, DNA duplexes were formed. The stability of the DNA duplexes was affected by the GC content, length, and sequence arrangement of the PCR products. Thus, the fluorescence signal of the dsDNA dye SYBR Green I (melting curve) was different and could also be used to specifically identify PCR products. Melting curve analysis is very similar to high resolution melting (HRM, a very popular biotechnology method for screening point mutations in clinical research) but more convenient and melting curve analysis can be performed in common real-time PCR systems rather than a special HRM analysis instrument. In this study, we combine DNA barcoding and melting curve analysis to authenticate saffron and its adulterants. Bar-MCA consists of a single step performed in a closed-tube procedure. It takes approximately 3 h to complete authentication. In contrast, sequencing requires more time (at least two working days) and costs approximately 10-fold more [45]. Melting curve analysis may also have considerable authenticating capacity and be able to differentiate samples at the genus or species level based on the *T*_*m*_ of specific PCR products [46]. One minor drawback is the need to obtain a melting curve database to serve as a standard when identifying unknown samples [45]. However, the high-throughput multiplex analysis capacity of real-time PCR systems and the low costs involved permit the simultaneous running of unknown samples and selected standards in each analysis.

In the present study,* trnH-psbA* was selected to authenticate the saffron and detect its adulterants by barcoding melting curve analysis. The plastid* trnH-psbA* intergenic spacer, which was proposed by Kress et al. [36] and incorporated into the core barcode for seed plants, is the most variable plastid region in angiosperms and is easily amplified across a broad range of land plants. For high polymorphism in sequence length and arrangement, the GC content would be different and affect the *T*_*m*_. Therefore, the *T*_*m*_ could be predicted by the analysis sequence from public databases, such as NCBI (National Center for Biotechnology Information), BOLD (Barcode of Life Data System) [47], or MMDBD (Medicinal Materials DNA Barcode Database) [48]. We obtained the* trnL-F*,* trnH-psbA*,* rbc*L,* mat*K, and ITS2 barcode sequences for saffron and its adulterants from public databases. We then aligned and unified the sequences with the clustalW program using the BioEdit software to unify the sequences from different labs that used different primers. When the *T*_*m*_ was calculated by OligoCalc, the *T*_*m*_ values for all 5 barcodes were different for saffron and its adulterants.* mat*K presented the largest temperature difference between* H. fulva* and* D. morifolium*.* trnL-F* had the maximum temperature difference between* N. nucifera* and* Z. mays* (Table 3). However, the predicted *T*_*m*_ results indicated that* mat*K is not able to differentiate* Hemerocallis fulva* from saffron because its small differences in *T*_*m*_.* trnL-F* cannot easily discriminate saffron and* D. morifolium*. ITS2 cannot distinguish saffron from the adulterant* C. officinalis*. Only* trnH-psbA* could differentiate saffron from its adulterants and distinguish the adulterants from each other based on the predicted *T*_*m*_. The excellent performance may be related to its large variation in sequence arrangement. Previous work has shown that in terms of interspecific divergence of barcodes, ITS2>*trnH-psbA*>*mat*K and* trnL-F*>*rbc*L [39, 49]. Although ITS2 has the highest discriminatory power by DNA barcoding, the melting temperature is small within saffron and its adulterants. This result may be due to the lack of sequence length variation between the samples when amplified with the ITS2/ITS3 primer pair. Nearly all of the samples produce a PCR product of approximately 500 bp, making changes in the *T*_*m*_ result that are based on sequence length difficult to detect. High polymorphisms in the same sequence may have compensatory effects at a symmetrical location in the sequence, producing only a minimal difference in *T*_*m*_, which would be difficult to detect by a conventional real-time PCR system. In contrast, the* trnH-psbA* fragment offers relatively high sequence polymorphisms and high sequence variations (Table 3) and affects the *T*_*m*_ due to length and GC content, which can generate a greater range of *T*_*m*_ differences. The results demonstrate that* trnH-psbA* may have a better discrimination capacity based on the melting temperature.

The *T*_*m*_ predictions for saffron and its adulterant are similar to the experimental *T*_*m*_ results. There is an approximately 2°C maximum deviation between the theoretical values and the experiment result for the adulterant* D. carota*, indicating that the *T*_*m*_ calculated from the sequence can serve as a preliminary reference in determining which primers for screening. Barcodes with large *T*_*m*_ differences could be used to prioritize confirmation experiments to reduce the experimental work.

## 5. Conclusion

Saffron is a very expensive medicinal spice product and is often adulterated by incorporation of materials such as* C. tinctorius* and* C. officinalis* flowers,* Hemerocallis* L. petals,* D. carota* fleshy root, C.* longa* rhizomes,* Z. may*, or* N. nucifera* stigmas. The real-time PCR-based method described in the present study is a powerful rapid and sensitive nonsequencing authentication technology that can detect adulterants in traded saffron. By amplification with the barcoding primer pair psbAF/trnHR and performing melting curve analysis, saffron was distinguished from the adulterants or detected in mixtures based on its *T*_*m*_. This technique could detect the presence of an expected plant material and adulterant materials in close-tube reactions. The method could be applied to other medicinal plants contaminated with adulterants.

## Figures and Tables

**Table 1:** The species origin, collect area, sample batch numbers, and identifier of saffron and its adulterants in the study.

Sample vochmen	Species	Origin	Identifier
Cs1	*Crocus sativus *	Anguo herb market, Hebei	Yan Jin
Cs2		Anguo herb market, Hebei	Yan Jin
Cs3		Iran BADIEE (Batch 20120110)	Yan Jin
Cs4		Iran BADIEE (Batch 20111018)	Yan Jin
Cs5		Iran BADIEE (Batch 20121120)	Yan Jin
Cs6		Tongrentang pharmacy (Batch A1320060920)	Yan Jin
Cs7		Tongrentang pharmacy (Batch A20120306101002768)	Yan Jin
Cs8		Tongrentang pharmacy (Batch A20111126101002768)	Yan Jin
Cs9		Tongrentang pharmacy (Batch A20100420001004968)	Yan Jin
Cs10		Tongrentang pharmacy (Batch A20120402200250329)	Yan Jin
Cs11		Bozhou herb market, Anhui	Yan Jin

CsA1	unknown	Anguo herb market, Hebei province	—
CsA2		Shanghai traditional Chinese medicine Co., Ltd.	—
CsA3			—
CsA4			—
CsA5			—
CsA6		Tongrentang pharmacy	—

Dm1	*Dendranthema morifolium *	TianHongJiSheng pharmacy	Yan Jin
Dm2		Jinyaotang pharmacy	Yan Jin
Dm3		Jinglongtang pharmacy	Yan Jin
Dm4		Jin xiang pharmacy	Yan Jin
Dm5		BoZhou herb market (2013)	Yan Jin
Dm6		BoZhou herb market (2012)	Yan Jin

Co1	*Calendula officinalis *	BoZhou herb market	Yan Jin

Ct1	*Carthamus tinctorius *	TianHongJiSheng pharmacy	Yan Jin
Ct2		JinYaoTang pharmacy	Yan Jin
Ct3		JinXiang pharmacy	Yan Jin
Ct4		BoZhou herbal market	Yan Jin

Nm1	*Nelumbo nucifera *	TianHongJiSheng pharmacy	Yan Jin
Nm2		JinYaoTang pharmacy	Yan Jin
Nm3		JinXiang pharmacy	Yan Jin
Nm4		JingLongTang pharmacy	Yan Jin
Nm5		Bozhou herbal market	Yan Jin

Dc1	*Daucus carota *	DongZhiMen super market	Yan Jin

Zm1	*Zea mays *	DongZhiMen super market	Yan Jin
Zm2		TianHongJiSheng pharmacy	Yan Jin
Zm3		JinYaoTang pharmacy	Yan Jin
Zm4		JingLongTang pharmacy	Yan Jin
Zm5		JinXiang pharmacy	Yan Jin

Hf1	*Hemerocallis fulva *	Bozhou herbal market	Yan Jin

**Table 2:** The primers sequence and PCR reaction in this paper.

Markers	Primer	Sequences 5′ to 3′	Real-time PCR reaction conditions
*trnH-psbA *	psbAF	GTTATGCATGAACGTAATGCTC	50°C 2 min; 95°C 30 sec; 95°C 5 sec, 58°C 34 sec, 40 cycles
	trnHR	CGCGCATGGTGGATTCACAATCC	

*rbc*L	1F	ATGTCACCACAAACAGAAAC	50°C 2 min; 95°C 30 sec; 95°C 5 sec, 60°C 34 sec, 40 cycles
	724R	TCGCATGTACCTGCAGTAGC	

ITS2	ITS2	ATGCGATACTTGGTGTGAAT	50°C 2 min; 95°C 30 sec; 95°C 5 sec, 60°C 34 sec, 40 cycles
	ITS3	GACGCTTCTCCAGACTACAAT	

*matK *	3F	CGTACAGTACTTTTGTGTTTACGAG	50°C 2 min; 95°C 30 sec; 95°C 5 sec, 50°C 10 sec, 54°C 34 sec, 40 cycles
	1R	ACCCAGTCCATCTGGAAATCTTGGTTC	

*trnL-F *	trnL	GGAAATCGGTAGACGCTACG	50°C 2 min; 95°C 30 sec; 95°C 5 sec, 60°C 34 sec, 40 cycles
	trnF	ATTTGAACTGGTGACACGAG	

**Table 3:** Predicted melting temperature (*T*_*m*_) of saffron and its adulterants from the sequence based on public database NCBI.

Species	Predicted *T*_*m*_ (°C)					*trnH-psbA *		
	*mat*K	*rbc*L	*trnL-F *	ITS2	*trnH-psbA *	Size (bp)	GC%	*T*_*m*___Exp__ (°C)
*Crocus sativus *	82.76	Not mentioned	83.54	90.84	83.38	609	37	81.92
*Dendranthema morifolium *	84.41	85.68	83.62	89.04	80.82	436	27	79.62
*Calendula officinalis *	83.69	87.23	83.94	90.79	81.31	476	27	79.92
*Carthamus tinctorius *	84.31	86.04	84.30	89.86	80.66	441	27	80.10
*Nelumbo nucifera *	84.28	86.12	89.73	89.52	80.11	359	27	80.58
*Hemerocallis fulva *	82.74	85.51	83.50	Not mentioned	83.95	669	35	84.78
*Zea mays *	Not mentioned	81.03	82.50	89.19	84.46	616	38	84.33
*Daucus carota *	84.11	86.12	83.71	88.73	83.86	315	38	81.08

Melting temperature of *trnL-F*, *trnH-psbA*, *rbc*L, *mat*K, and ITS2 barcodes was predicted by an online OligoCalc program, amplicon size, and GC content was also calculated and the experiment *T*_*m*_ was present. *T*_*m*___Exp__ represent the melting temperature from Bar-MCA result.
